# Measurement of healthy ageing

**DOI:** 10.1093/ageing/afad118

**Published:** 2023-10-30

**Authors:** Muthoni Gichu, Rowan H Harwood

**Affiliations:** Division of Geriatric Medicine, Ministry of Health, Nairobi, Kenya; School of Health Sciences, Queen's Medical Centre, Nottingham NG7 2HA, UK

**Keywords:** health, ageing, measurement, older people

## Key Points

- Decade of healthy ageing
- Monitoring
- Evaluation
- Healthy ageing

##  

The United Nations declared 2021-2030 as the Decade of Healthy Ageing. World Health Organisation (WHO) is leading in the implementation and monitoring of the decade. Monitoring is needed to drive action, provide governance and enable evaluation.

The concept of healthy ageing, or successful ageing, is controversial. Some have defined it as reaching older age without disease or disability. But this has been challenged as narrow, exclusive and potentially discriminatory [1, 2]. Many degenerative conditions are unavoidable, and it is prejudicial to label those afflicted by illness or injury as ‘unsuccessful’. Recent efforts reframe the goal of chronic disease management as ‘living well’ with the condition. In the face of frailty, multimorbidity and disability people can live worthwhile, fulfilling, successful lives, with a good quality of life; older people may recognise this even when their doctors do not [3]. Disease and disability prevention are important, but we can and should strive to promote and support healthy lives even after they have occurred.

In its 2015 World Report on Ageing and Health, WHO defined healthy ageing as ‘the process of developing and maintaining the functional ability that enables wellbeing in older age’ [4]. Further, healthcare and public health have traditionally focused on individual diagnoses, a problem for older people, who often have multimorbidity. As a consequence, they may experience fragmented care, contact with multiple specialists, iatrogenesis, polypharmacy and a lack of regard for person-centred priorities. Not all diagnoses have the same impact on function and quality of life; multiple diagnoses can have a cumulative and interacting effect. In addition, mental disorders, social vulnerability and the environment have a disproportionate effect on the well-being of older people. Healthy ageing might be seen as perpetuating over-simplification, but it highlights the centrality of function, and reminds us that the ultimate goal is ‘wellbeing’. Function—the ability to do things that are necessary or wanted—integrates the effects of both multimorbidity and context.

‘Function’ can be nebulous, however, and we need a working definition. Mobility is central to everyday functioning but does not define it. The recent SPRINTT trial study group proposed ‘inability to walk 400m’ as a universal criterion for defining mobility disability. This is reasonable but is essentially arbitrary [5]. Further, many empirical scales have been developed to measure activities of daily living (ADLs), and they have been used to indicate functionality since the 1980s. Failure to standardise has, however, led to problems at the public health level, for example in determining if ‘health span’ is increasing at the same rate as lifespan, or whether ‘compression of morbidity’ is occurring [6, 7]. Few ADL scales have been validated in low- and middle-income countries, further limiting their usefulness. A new driver for standardisation is the potential for research from large datasets derived from electronic patient record systems, which has been constrained for older people by the absence of relevant functional variables [8].

WHO led previous attempts to systematically develop frameworks describing the consequences of ill-health. The International Classification of Impairments, Disabilities and Handicaps [9], then the International Classification of Disability, Functioning and Health [10], brought advances in conceptual understanding, but were complex, and have been difficult to operationalise. Alternative systems, such as the InterRAI suite of standardised measures, have developed pragmatically for application in particular settings [11].

The World Report on Ageing and Health defines functional ability as the ‘capability that enables people to be and do what they have reason to value’, with domains of meeting basic needs; ability to learn, grow and make decisions; being mobile; building and maintaining relationships; and contributing to society. Functional ability results from an interaction between basic components of ‘intrinsic capacity’ (mobility, cognition, sensory function, psychology and vitality) and the physical and social environment in which an individual lives [4]. This represents a useful summary of previous frameworks. It has been validated using epidemiological datasets from the United Kingdom and China [12, 13], but as yet lacks widespread empirical application. Further experience will show if the model successfully includes all the important determinants of well-being in older age, whilst being simple enough for clinical use and incorporation into large, multinational population surveys, such as the WHO World Health Survey.

The framework represents new thinking, a parsimonious set of dimensions, framed in positive terms rather than as deficits, which are likely to have the greatest impact on well-being. But it raises interesting measurement problems, such as defining the domains of ‘positive psychology’ or ‘vitality’ and then identifying or developing appropriate measures to quantify them. Even familiar dimensions, such as mobility, raise questions of detail, including scale (dexterity versus gross mobility and mobility in challenging environments), accommodation of assistive devices and technologies (mobility aids, wheelchairs and vehicles) and the role of human help. Healthcare has long privileged independent function, when the experience of people living with disabilities and their families is more in terms of adaptation, assistance and supported inclusion, which are difficult to measure. The set of systematic reviews presented in this supplement represents a huge effort to define what is already known, what is available and where the gaps are that will determine future research and development priorities.

Healthcare for older adults operates at multiple levels. Ideally, we prevent illness, injury and complications. When ill-health occurs, there is need for a timely diagnosis and management of conditions. Interventions could be through medical treatment, surgery, rehabilitation therapies or environmental adaptation including culture and attitudes. Or avoiding or stopping harmful therapies [14]. There may be a need for more or different human interactions or practical assistance from families or professionally provided. The domains of intrinsic capacity will indicate the targets for medical or therapy assessment and treatment whilst those of functional ability will define its success.

The aim of the Decade of Healthy Ageing is universal. Older people need to be planned for regardless of societal development or wealth. Middle- and lower-income countries in particular are ageing rapidly and need to identify practical and applicable approaches to optimising health in older age. A system based on intrinsic capacity may help deliver that [15]. A universal metric of healthy ageing will support and advance the goal of a healthy old age for all and needs to be accompanied by necessary actions.
